# The impact of the relationship between government and pharmaceutical enterprises on social contribution during the public health emergency: an empirical study

**DOI:** 10.3389/fpubh.2025.1494922

**Published:** 2025-02-18

**Authors:** Qian Zhuang, Huan Wang, Qiqi Bai, Jingwen Liang

**Affiliations:** School of International Pharmaceutical Business, China Pharmaceutical University, Nanjing, China

**Keywords:** social contribution, government-enterprise alignment, public health emergency, communication mechanism, party organization

## Abstract

**Introduction:**

During the COVID-19 pandemic, vaccines and specific drugs are seen as indispensable solutions to ending or responding to the pandemic, and pharmaceutical enterprises are in the spotlight. The Chinese government has made active efforts to guide pharmaceutical enterprises to make appropriate social contribution during the public health emergency. This study explores how government-enterprise relationship promotes this process.

**Methods:**

Using the financial and textual data of China's listed pharmaceutical companies and policy data from the official website of the Chinese health-related government departments, this study drew the social contribution through text analysis, and established the response index of pharmaceutical companies to the government—the government-enterprise alignment index (*GE_Ali*) based on the formula of elasticity for reference. Then a series of regressions are used to do the empirical tests.

**Results:**

This study found the more responsive pharmaceutical companies were to government, the greater their contribution to society during the pandemic, mainly through increasing the intensity of drug R&D, production and promotion, and the good communication mechanism between the two formed a mediating effect.

**Discussion:**

The nature of state ownership, the presence of embedded party organizations, and the location in the provincial capital city had significant effects on the realization of a high level of government-enterprise alignment positively affecting the social contribution. This study confirms that the Chinese government has made enterprises a part of social governance, which is a global hotspot, through the embedding of party organizations. It also indicates that the government needs to re-recognize its key role in shaping social contribution, especially in distinguishing its responsibilities between normal and emergency situations.

## 1 Introduction

At the beginning of 2020, the COVID-19 epidemic broke out. The Chinese government actively responded, and then the world also adopted measures such as isolation, social distancing, wearing masks in public places, and coordinated rationing of medical resources. The huge impact of COVID-19 on businesses is seen as an incentive for companies to fulfill their true corporate social responsibility (CSR) by contributing to society during the pandemic (1). The widespread impact of the pandemic crisis at the societal level is driving companies to focus more on authentic CSR practices and a genuine commitment to solving social problems (2).

Specific CSR practices in certain industries during the pandemic crisis have received attention. The development and distribution of vaccines is considered an indispensable solution to ending or responding to a pandemic, so pharmaceutical companies are in the spotlight, and issues related to the equity of global distribution of vaccines are the most prominent CSR of large pharmaceutical companies. A study of six vaccine developers^1^ who had passed the Emergency Use Listing (EUL) issued by the World Health Organization (WHO) by August 2021 explored the CSR aspects of global distribution of vaccines during a pandemic, including whether the product development process took into account the needs of people in low- and middle-income countries; whether it provides fair access to its products, such as fair pricing, waiver of intellectual property rights and product donations; whether clinical trial data is transparent and accountable (3). The Chinese case is unique in that community organizations play an important role as grassroots units, and the state plays a key role in shaping corporate social engagement and getting companies to work with the government during the emergency. Pharmaceutical companies are more deeply involved in community anti-epidemic activities as their products, services, and expertise are related to protecting human health and saving lives (4).

The COVID-19 pandemic has led to better theorizing about how companies can be part of social governance (5). It has repositioned government as a key player in addressing major challenges (6), and the resurgence of government in CSR research challenges the assumption that companies can voluntarily deliver public goods and close governance gaps, as there is a growing recognition that these “soft” initiatives, such as industry self-regulation and voluntary agreements, are insufficient to address the world's most pressing social and ecological challenges (42). On the other hand, levels of altruism and corporate solidarity tend to increase during national and global disasters, which leads to higher altruistic engagement by companies (7, 8). The “two-way efforts” between government and enterprises make the relationship between them closer.

During the epidemic period, the Chinese government has formulated anti-epidemic strategies at the national level, utilizing the resources of various domestic organizations to fight against the epidemic. Not only the Communist Party apparatus and the government at all levels but also business enterprises, non-profit organizations, and ordinary people have participated in the national project to protect public health (9). The purpose of enterprise production is to make profits, but when the whole community is facing the crisis of epidemic prevention and control, enterprises need to shoulder the necessary CSR. In this paper, we refer to the specific CSR in responding to social crises as the social contribution, and try to make it clear in the context of the public health emergency, and explore the impact and mechanism of “corporate response to the government” on the social contribution so as to deeper understand enterprises as part of social governance.

## 2 Theoretical analysis and hypothesis development

### 2.1 Government-enterprise relationship and social contribution

Emergency public health events have changed the priority of corporate stakeholder identification to some extent. Due to legitimacy, power and urgency, the government has been raised to the optimal level of corporate identification (6), highlighting the importance of government-enterprise relationship in fulfilling social contribution. CSR is shaped in the relationship between business and government (10). Today, companies collaborate with governments or business associations to share resources. In this partnership, governments provide financial assistance and regulation, while companies support their network relationships, employee welfare, and knowledge output. There is evidence that collaboration between government and enterprises can benefit society. Especially in emergency situations such as disasters, governments and enterprises carry out disaster response, recovery and mitigation through financial support, donations, planning and other initiatives (11). Based on this collaboration, the government shapes, selects and guides CSR conscious companies to undertake social contribution during the COVID-19 pandemic (12). Companies with strong political awareness are more open and receptive to social issues. They will change their corporate governance structure to improve the transparency of their political activities, make it easier for internal and external supervisors to obtain corporate information, and better hold stakeholders accountable for problematic social behaviors. Politically responsible companies are more likely to act socially during the COVID-19 pandemic (13).

The relationship between enterprises and government has always been the focus of research on CSR practices in emerging markets (14). The emergency scenario of important public outburst increases the interaction between enterprises that master professionalism and connect the existing industrial chain and the government (15–17). During the COVID-19 pandemic, the medical supplies market is in short supply, qualified suppliers are limited, and time constraints and cognitive constraints are more obvious. Single, non-competitive contracts and cooperation are often adopted, requiring multi-faceted government intervention to help enterprises reduce the impact of market disruption and negotiate to meet emerging social needs. Enterprises should take into account the present, and balance long-term production capacity with supply and demand fluctuations, customer relationship maintenance, enterprises rely on the government for a wider range of long-term. Enterprises will choose to take the initiative to cooperate with the government to enhance their legitimacy and reputation on the basis of social contribution commitment (18). In the early stage, a good government-enterprise cooperation relationship or an enterprise's response to the government relationship can effectively solve the problem of information asymmetry, and help enterprises quickly identify social contribution strategies and motives oriented to public services and public interests, so as to quickly realize resource allocation and resource complementarities under the epidemic situation, and alleviate the social crisis. Pharmaceutical companies have an ethical obligation to ensure that vaccines are developed and distributed fairly in a way that optimizes health and economic outcomes (19). The government will have a high degree of trust in pharmaceutical companies with a good basis for cooperation, and actively negotiate with them on the pricing, listing and distribution of vaccines, so as to achieve a fair and effective distribution of vaccines in society.

Accordingly, the following hypotheses are proposed in this paper:

Hypothesis 1 (H1): In the context of the public health emergency, a higher level of government-enterprise alignment has a positive impact on the social contribution of pharmaceutical enterprises.

### 2.2 Government-enterprise relationship, communication mechanism, and social contribution

In order to cope with the epidemic crisis, the Chinese government has adopted the strategy of penetrative cooperation (20). Government and enterprises are at different levels of information dissemination and have different communication resources (21). Certain industries and organizations are more exposed to the public spotlight in a crisis, and their strategies and decisions are more likely to be questioned by stakeholders, as well as criticized by the public, such as promoting non-essential items during the pandemic (44). Companies need to gain public trust through broader information sources, such as international or national media sources, to enhance legitimacy and reputation. At the same time, governments also need more micro information resources to solve local problems or events. From the perspective of reflexivity, good communication mechanisms can improve the collective ability to plan, act, reflect, and solve problems (22, 23). Communicate and interact promote reaching agreement on action to solve social crises. The public has clear expectations of certain industries, and the communication of social contribution engagement is actually a set of strategies that respond to public interests and priorities. In such a serious crisis, the government also faces the gradual and incomplete understanding of the characteristics of the virus, the overall shortage of supply and the intensified market segmentation of the “lockdown” exacerbate the uncertainty of prevention and control materials, etc. (18). The impact of the epidemic is long-term, resulting in ever-changing demands, and the government's expectations do not match the information provided. Enterprises need to provide a wider range of more frequent communication to meet information needs.

Accordingly, the paper further proposes the following hypotheses:

Hypothesis 2 (H2): In the context of the public health emergency, a higher level of government-enterprise alignment positively impacts the social contribution of pharmaceutical enterprises through effective communication mechanisms.

## 3 Research design

### 3.1 Sample, data and methods

In this paper, Shanghai and Shenzhen A-share pharmaceutical listed companies from 2020 to 2022 are selected as research samples, and the pharmaceutical industry classification is based on the 2021 version of Shenyinwanguo Industry Classification. The number of policies published on the official websites of the National Health Commission and the National Medical Products Administration was used. Since the policies issued by the National Healthcare Security Administration focus on the medical security system, mainly involving basic medical insurance and medical insurance fund, they were not included. The social contribution degree is obtained by text analysis of the company's annual report and CSR report, and the government-enterprise relationship is obtained by developing the index calculation formula of “the degree of an enterprise's response to the government” based on the formula of elasticity for reference, which is defined as government-enterprise alignment. In addition, in order to avoid the influence of outliers, this paper carried out 1% tailing treatment for all continuous variables, and finally obtained 316 sample observations.

### 3.2 Measurement of variables

#### 3.2.1 Social contribution

Through text analysis of keywords and key statements, the social contribution indicators of pharmaceutical enterprises during the public health emergency were developed based on the Issue Salience (8), mainly focusing on three aspects: drug R&D, production and promotion, public welfare donation, and supply security, each of which corresponds to different contribution behaviors, as shown in Table 1. If the pharmaceutical enterprise has these social contribution behaviors in the epidemic (disclosed in the annual report or CSR report), it will be assigned a value of 1 under the corresponding secondary index, otherwise it will be 0, and finally all the values will be added to get the overall social contribution of the pharmaceutical enterprise. The larger the index value, the greater the enterprise's contribution to society during the public health emergency.

**Table 1:** Social contribution indicator system.

**Primary indexes**	**Secondary indexes**	**Interpretation of secondary indexes (1 point for one of them)**
Drug R&D, production and promotion (Medical Needs)	(a) Drug R&D and production	• R&D and production of drugs and vaccines for the prevention and control of public health events, e.g. conducting clinical trials for relevant indications during the pandemic • Participate in the global synchronous R&D of drugs (including participating in the cooperation of foreign pharmaceutical companies to develop preferred drugs, vaccines, etc.; participating in international multi-center clinical trials, etc.)
	(b) Development of auxiliary diagnostic instruments and protocols	• R&D and production of medical devices for auxiliary treatment and diagnosis, development and design of diagnosis and treatment plans, e.g. development of nucleic acid test kits, probes, primers, cupping processing systems, etc.; provision of plasma therapy, etc.; during the pandemic
	(c) Sharing of diagnostic and treatment programs and technical resources	• Sharing of diagnosis and treatment plans: exchanging and sharing of diagnosis and treatment plans, e.g. conducting online expert seminars; providing of Traditional Chinese Medicine diagnosis and treatment plans; sharing of clinical research data on past relevant cases, etc. • Sharing of technical resources: disclosure of relevant patent information, lowering technical barriers, so that generic drugs or related products can be promoted quickly • Undertaking logistics and transportation: undertaking the logistics and transportation of drugs, vaccines, biological agents, instruments and other products, to ensure the correctness, standardization, timeliness and process of special product transportation
Public donation (Donation)	(a) Donation of medical supplies	• Donation of medical supplies, e.g., medicines, medical masks, protective suits, ventilators, etc., during the epidemic
	(b) Medical staff support	• Professional medical team and relevant volunteer support
	(c) Medical information guide	• Publicity and guidance on protection knowledge related to public health events, e.g., the collation of epidemic prevention information during the epidemic; publicity and promotion of epidemic prevention knowledge; reducing public bad mood, etc.
Supply security (Supply)	(a) Supply of medical products	• Guaranteeing the accessibility of medical products of the enterprise's pipeline
	(b) Staff health and equipment safety	• Ensure the health status of employees, e.g., some posts working offline during the epidemic; staff temperature measurement, epidemic prevention knowledge training, psychological counseling, etc., after the resumption of work and production, etc. • Ensure the normal operation of equipment, e.g., the elimination process when operating the equipment; the equipment is adjusted to the normal use mode; laboratory biosafety inspection and other hidden dangers investigation, etc., after the resumption of production and work, etc.
	(c) Coordination and scheduling of medical resources	• Taking responsibility for treatment and transfer medical resources, e.g., transforming private hospitals into designated isolation hospitals during the epidemic; coordinating and scheduling of instruments, beds and other medical resources, etc.

**Figure 1:**
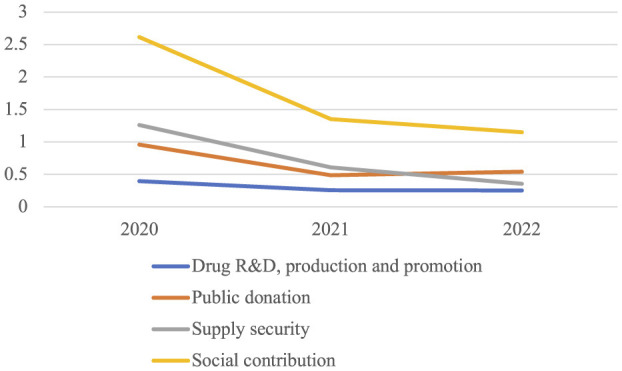
Trend chart of scores of social contribution and three dimensions.

The total score of each enterprise's social contribution ranges from 0 to 9. From Figure 1, it can be seen that the average score of the enterprise's social contribution does not exceed 3, which can be considered very low. From a trend perspective, drugs R&D, production and promotion dimension has remained almost unchanged over the past 3 years, while the other two dimension and social contribution in total have shown the highest scores in 2020, the 1^st^ year of the outbreak, and downward trend from 2021 to 2022.

#### 3.2.2 Government-enterprise alignment

Through the common concern of pharmaceutical enterprises and relevant government departments—disease prevention and control, that is, the possibility of achieving the same goal, the index of government-enterprise alignment (*GE_Ali*) is established. The index of *GE_Ali* = Changes in enterprise's attention in the field of disease/Changes in government's attention in the same field of disease, where ICD-11 code is used to judge the field of disease, the company's attention in the field of disease is expressed by the sales revenue in the annual report, and the government's attention in the field of disease is expressed by the number of policies issued by relevant government departments. This formula draws inspiration from the formula for calculating elasticity in economics, and shown as follows:

Changes in corporate focus in the field of disease = $\overset{n}{\underset{i=2}{\sum }}\frac{\frac{SR_{i}\;this\;year\;}{SR\;this\;year}-\frac{SR_{i}\;last\;year}{SR\;last\;year}}{\frac{SR_{i}\;last\;year}{SR\;last\;year}}$

Changes in government concern in the field of disease = $\overset{n}{\underset{i=2}{\sum }}\frac{\frac{GP_{i}\;this\;year\;}{GP\;this\;year}-\frac{GP_{i}\;last\;year}{GP\;last\;year}}{\frac{GP_{i}\;last\;year}{GP\;last\;year}}$

In particular, when *i* = 1, the following formula is used

Changes in corporate focus in the field of disease = $\frac{SR\;this\;year-SR\;last\;year}{SR\;last\;year}$

Changes in government concern in the field of disease = $\frac{GP\;this\;year-GP\;last\;year}{GP\;last\;year}$

in the formular, *SR* represents the sales revenue of the company's drugs, *GP* represents the number of policies issued by the government, *i* represents the field of prevention and treatment of a disease.

**Figure 2:**
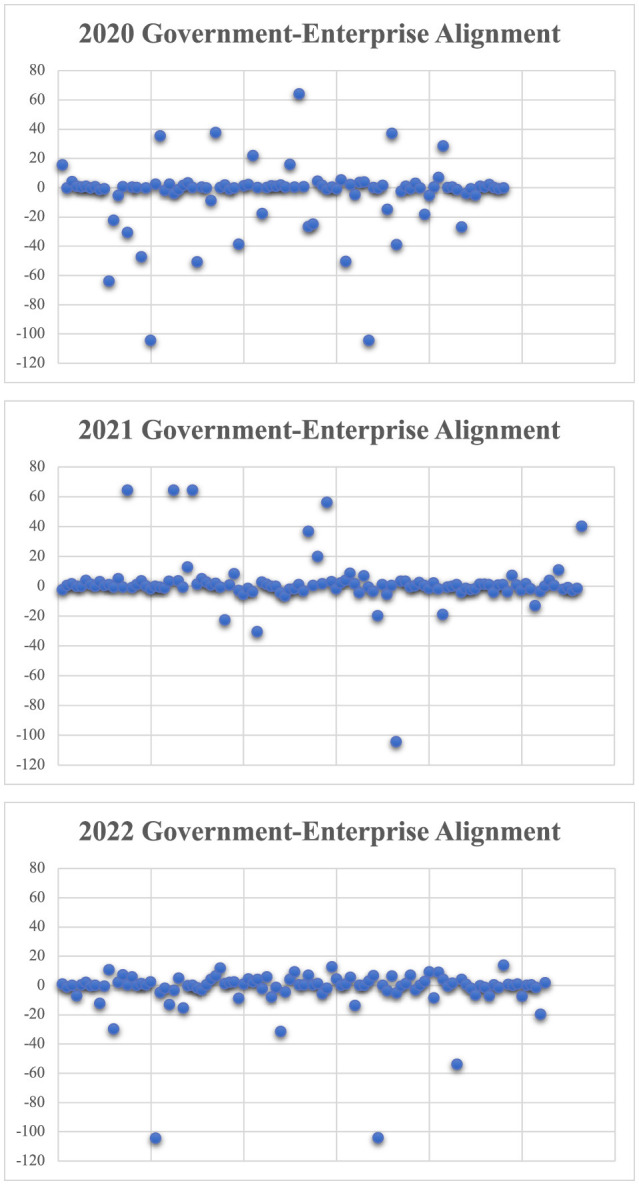
Scatter plot of government-enterprise alignment of 2020, 2021, 2022.

From Figure 2, it can be seen that the vast majority of enterprises' response to the government fluctuates around 0 value. In the 3 years of the epidemic, the number of deviations has also decreased, indicating that enterprises are increasingly concerned about the guidance of government policies.

#### 3.2.3 Control variables

Drawing on the research of Kim et al. (24), Chang et al. (25), Kang (26) and Chen et al. (27), Financial Slack (*Slack*), Ownership Concentration (*Top*1), Asset-liability Ratio (*Lev*), Nature of Ownership (*SOE*), Asset Size (*Size*), and Age of Enterprise (*Age*) were selected as control variables. The variables are defined in Table 2.

**Table 2:** Definition of variables.

**Type of variables**	**Name of variables**	**Symbol of variables**	**Measurement of variables**
Dependent variables	Social contribution	*SC_Total*	Scoring through text analysis
	Drug R&D, production and promotion	*Medical Needs*	The score of drug R&D, production and promotion part in social contribution
	Public donation	*Donation*	The score of public donation part in social contribution
	Supply security	*Supply*	The score of supply security part in social contribution
Independent variable	Government-enterprise alignment	*GE_Ali*	Changes in enterprise's attention in the field of disease/Changes in government's attention in the same field of disease
Control variables	Financial slack	*Slack*	Cash and cash equivalents/total assets
	Ownership concentration	*Top*1	The shareholding ratio of the largest shareholder of the company
	Asset-liability ratio	*Lev*	Total liabilities/total assets
	Nature of ownership	*SOE*	1 stands for state-owned enterprises, 0 for non-state-owned enterprises
	Asset size	*Size*	ln (total assets)
	Age of enterprise	*Age*	Date of Establishment

### 3.3 Modeling

To investigate the impact of government-enterprise alignment on social contribution, the following multiple linear regression model is constructed for empirical analysis.


(1) $$\begin{array}{r} {SC\text{\_}Total}_{it}=\alpha_{0}+\alpha_{1}{GE\text{\_}Ali}_{it}+\alpha_{i}Control_{it}\\ +SUBINDUSTRY+YEAR+\varepsilon_{it}\; \end{array}$$

(2) $$\begin{array}{r} MedicalNeeds_{it}=\alpha_{0}+\alpha_{1}{GE\text{\_}Ali}_{it}+\alpha_{i}Control_{it}\\ +SUBINDUSTRY+YEAR+\varepsilon_{it} \end{array}$$

(3) $$\begin{array}{r} Donation_{it}=\alpha_{0}+\alpha_{1}{GE\text{\_}Ali}_{it}+\alpha_{i}Control_{it}\\ +SUBINDUSTRY+YEAR+\varepsilon_{it} \end{array}$$

(4) $$\begin{array}{r} Supply_{it}=\alpha_{0}+\alpha_{1}{GE\text{\_}Ali}_{it}+\alpha_{i}Control_{it}\\ +SUBINDUSTRY+YEAR+\varepsilon_{it} \end{array}$$

Models (1) to (4) aim to verify the research hypothesis of government-enterprise alignment on the social contribution of enterprises in the context of the public health emergency, and explore the impact of government-enterprise alignment on three dimensions of social contribution: medical needs, donation, and supply. *Control*_*it*_ involved in the model refers to control variables including Financial Slack (*Slack*), Ownership Concentration (*Top1*), Asset-liability Ratio (*Lev*), Nature of Ownership (*SOE*), Asset Size (*Size*), and Age of Enterprise (*Age*). In addition, this study also controlled for the fixed effects of sub industry (*SUBINDUSTRY*) and year (*YEAR*).

## 4 Empirical results

### 4.1 Descriptive statistical analysis

Table 3 reports the descriptive statistical results of the main variables in the pharmaceutical industry. From Table 3, it can be seen that the average and median values of the variable *SC_Total* reflecting the social contribution of enterprises are 1.668 and 1.000, respectively, with a standard deviation of 1.800. The maximum and minimum values are 8.000 and 0.000, indicating that there are significant differences in the degree of social contribution of sample enterprises during the sudden public health emergency, and the overall contribution level is relatively low.

**Table 3:** Descriptive statistics of main variables.

**Variables**	**Observation**	**Average value**	**Median value**	**Standard deviation**	**Minimum value**	**Maximum values**
*SC_Total*	316	1.668	1.000	1.800	0.000	8.000
*Medical Needs*	316	0.297	0.000	0.607	0.000	3.000
*Donation*	316	0.649	0.000	0.764	0.000	3.000
*Supply*	316	0.722	0.000	0.897	0.000	3.000
*GE_Ali*	316	−1.938	−0.033	18.940	−104.700	63.980
*Slack*	316	0.190	0.157	0.128	0.004	0.583
*SOE*	316	0.237	0.000	0.426	0.000	1.000
*Top*1	316	31.820	30.560	11.300	10.450	60.900
*Size*	316	22.410	22.310	1.061	20.530	25.400
*Age*	316	22.880	23.000	5.197	10.000	35.000
*Lev*	316	0.342	0.315	0.184	0.040	0.870

The average and median values of the variable (*GE_Ali*) reflecting the degree of response of enterprises to the government are −1.938 and −0.033, respectively, with a standard deviation of 18.940. The maximum and minimum values are 63.980 and −104.700, indicating that there is a significant gap in the degree of response of enterprises to the government, and overall, the degree of response is also relatively low. In terms of controlling variables, the standard deviation of Ownership Concentration (*Top*1) and Age of Enterprise (*Age*) are both relatively large, indicating that the degree of corporate social contribution may be affected by these differences.

Table 4 reports the correlation coefficients between variables in the sample enterprises. The social contribution is significantly positively correlated with the index of *GE_Ali* at the 10% level, indicating that a higher level of *GE_Ali* is beneficial for the improvement of the social contribution of pharmaceutical companies during the public health emergency, which preliminarily verifies hypothesis 1. The Nature of Ownership (*SOE*) and Asset Size (*Size*) are significantly positively correlated with social contribution (*SC_Total*) at the 1% level, indicating that state-owned pharmaceutical companies have a higher level of social contribution, and the larger the asset size, the higher the social contribution of pharmaceutical companies during the public health emergency. There is a significant positive correlation between Ownership Concentration (*Top1*) and social contribution (*SC_Total*) at the 10% level, indicating that in the event of a public health emergency, the higher the shareholding ratio of the largest shareholder and the greater their influence on pharmaceutical companies, the greater the social contribution of pharmaceutical companies. There is a significant positive correlation between the age of a company (*Age*) and its social contribution at the 5% level, indicating that the longer the company has been established, the more favorable it is for the company to make social contribution during the public health emergency.

**Table 4:** Correlation analysis of main variables.

	***SC_Total***	***Medical needs***	***Donation***	***Supply***	***GE_Ali***	***Slack***	***SOE***	***Top1***	***Size***	***Age***	***Lev***
*SC_Total*	1										
*Medical needs*	0.626^***^	1									
*Donation*	0.817^***^	0.246^***^	1								
*Supply*	0.886^***^	0.368^***^	0.621^***^	1							
*GE_Ali*	0.101^*^	0.178^***^	0.043	0.045	1						
*Slack*	−0.006	0.130^**^	0.002	−0.102^*^	0.035	1					
*SOE*	0.219^***^	0.008	0.198^***^	0.265^***^	0.035	−0.077	1				
*Top*1	0.096^*^	0.130^**^	0.078	0.038	−0.020	0.192^***^	0.029	1			
*Size*	0.353^***^	0.300^***^	0.270^***^	0.276^***^	0.055	−0.149^***^	0.336^***^	0.075	1		
*Age*	0.144^**^	0.046	0.157^***^	0.124^**^	0.024	−0.146^***^	0.257^***^	−0.104^*^	0.342^***^	1	
*Lev*	−0.009	0.006	−0.019	−0.006	−0.078	−0.152^***^	0.258^***^	−0.123^**^	0.262^***^	0.216^***^	1

^*^, ^**^, and ^***^ indicate significance at the 10%, 5% and 1% levels respectively.

In addition, the results of a multicollinearity test on the variables are shown in Table 5, indicating that the variance inflation factor (VIF) is much smaller than 10, so it is considered that the model does not have multicollinearity.

**Table 5:** Multicollinearity test.

**Variables**	**VIF**	**Tolerance**
*Size*	1.300	0.772
*Age*	1.200	0.832
*SOE*	1.200	0.837
*Lev*	1.170	0.856
*Top*1	1.080	0.922
*Slack*	1.080	0.923
*GE_Ali*	1.020	0.983
Mean	VIF	1.150

### 4.2 Benchmark regression results

We use ordinary least squares method (OLS) for estimation (28). To fully explore the impact of government-enterprise relationship on social contribution in the context of the public health emergency, we analyze the three dimensions of medical needs, public welfare donations, and supply security in social contributions one by one. Table 6 reports the empirical results of models (1) to (4). The results showed a significant positive correlation (α = 0.011, *p* < 0.01) between government-enterprise alignment and their social contribution at the 1% level, indicating that as the response of enterprise to the government continues to increase, the social contribution of the pharmaceutical enterprise will also correspondingly increase during the public health emergency, verifying hypothesis 1. Moreover, there is a significant positive correlation (α = 0.006, *p* < 0.01) between government-enterprise alignment and the dimensions of drug R&D, production, and promotion in social contributions at the 1% level, indicating that during the public health emergency, higher levels of government-enterprise alignment mainly enhance the intensity of drug R&D, production, and promotion in the social contributions of pharmaceutical companies, but have no significant impact on the dimensions of public welfare donations and supply security in social contributions.

**Table 6:** Main effect regression results of government-enterprise alignment on the social contribution.

**Variables**	**(1)**	**(2)**	**(3)**	**(4)**
	***SC_Total***	***Medical Needs***	***Donation***	***Supply***
*GE_Ali*	0.011^***^	0.006^***^	0.002	0.003
	(0.004)	(0.002)	(0.002)	(0.002)
*Slack*	0.979	0.818^***^	0.343	−0.183
	(0.670)	(0.278)	(0.297)	(0.331)
*SOE*	0.483^*^	−0.151	0.204^*^	0.430^***^
	(0.260)	(0.092)	(0.115)	(0.124)
*Top*1	0.004	0.004	0.001	0.000
	(0.008)	(0.003)	(0.004)	(0.004)
*Size*	0.494^***^	0.174^***^	0.165^***^	0.155^***^
	(0.100)	(0.039)	(0.043)	(0.048)
*Age*	0.013	−0.003	0.010	0.006
	(0.020)	(0.007)	(0.008)	(0.009)
*Lev*	−1.309^**^	0.052	−0.651^***^	−0.711^***^
	(0.510)	(0.160)	(0.234)	(0.246)
*Constant*	−8.878^***^	−3.705^***^	−2.933^***^	−2.241^**^
	(2.083)	(0.880)	(0.926)	(1.004)
YEAR	YES	YES	YES	YES
SUBINDUSTRY	YES	YES	YES	YES
N	316	316	316	316
Adj R^2^	0.277	0.171	0.167	0.287

Robust standard deviations are shown in parentheses; ^*^, ^**^, and ^***^ indicate significance at the 10%, 5%, and 1% levels respectively.

### 4.3 Endogeneity

To address endogeneity issues, this study used the mean social contribution of other companies in the same industry (*SC_TotalAve*) as an instrumental variable and employed a two-stage least squares method for endogeneity treatment.

The first stage model of instrumental variable regression is:


(5) $$\begin{array}{r} {GE\text{\_}Ali}_{it}=\alpha_{0}+\alpha_{1}{SC\text{\_}TotalAve}_{it}+\alpha_{i}Control_{it}\\ +SUBINDUSTRY+YEAR+\varepsilon_{it} \end{array}$$

Table 7 presents the results of two-stage instrumental variable regression. The results of the first stage regression are shown in column (1) of Table 7, where the *SC_TotalAve* coefficient is significantly negative, rejecting the null hypothesis that there is no problem of insufficient identification. Therefore, there is a correlation between the instrumental variable and the endogenous variable. Meanwhile, in the weak instrumental variable test, the Cragg Donald Wald F statistic value is 5.2 × 10^4^, which is higher than the weak ID test critical value of 16.38 at the 10% level, indicating that the instrumental variable corresponding to the core variable is not a weak instrumental variable. The above indicates that the selection of instrumental variable is appropriate. The regression results in column (2) of Table 7 show that in the context of the public health emergency, the regression coefficient between government-enterprise alignment and social contribution is significantly positive, indicating that after controlling for a series of endogeneity issues, the conclusions still hold true.

**Table 7:** Regression results of the instrumental variable.

**Variables**	**(1)**	**(2)**
	***GE_Ali***	***SC_Total***
*GE_Ali*		0.012^***^
		(0.004)
*SC_TotalAve*	−99.632^***^	
	(1.624)	
Control variables	YES	YES
YEAR	YES	YES
SUBINDUSTRY	YES	YES
Cragg-Donald Wald F	5.2 × 10^4^	
N	312	312

Robust standard deviations are shown in parentheses; ^***^ indicate significance at the 1% level.

### 4.4 Robustness tests

To ensure the robustness of the empirical results, we conducted robustness tests from two aspects: replacing the empirical model and replacing variables.

#### 4.4.1 Replacing the empirical model

Considering the characteristics of the value of social contribution (some enterprises have a value of 0), which is a partially restricted dependent variable, the Tobit model is adopted for robustness testing, as shown in Table 8. The results show that using the Tobit model, government-enterprise alignment is significantly positively correlated with social contribution and drug R&D, production and promotion (α = 0.02, *p* < 0.01; α = 0.03, *p* < 0.01), and there is no significant impact on public welfare donations and supply security, which is consistent with the results in Table 8 and also verifies hypothesis 1.

**Table 8:** Robustness test - Tobit model.

**Variables**	**(1)**	**(2)**	**(3)**	**(4)**
	***SC_Total***	***Medical Needs***	***Donation***	***Supply***
*GE_Ali*	0.020^**^	0.031^***^	0.004	0.008
	(0.008)	(0.008)	(0.004)	(0.005)
*Slack*	1.209	2.869^***^	0.119	−0.961
	(1.114)	(0.949)	(0.669)	(0.780)
*SOE*	0.580^*^	−0.861^**^	0.292	0.794^***^
	(0.335)	(0.335)	(0.193)	(0.217)
*Top*1	0.010	0.013	0.003	−0.001
	(0.012)	(0.011)	(0.007)	(0.009)
*Size*	0.668^***^	0.609^***^	0.297^***^	0.324^***^
	(0.146)	(0.134)	(0.084)	(0.097)
*Age*	0.006	−0.022	0.032^*^	0.015
	(0.029)	(0.025)	(0.017)	(0.020)
*Lev*	−1.877^**^	0.354	−1.588^***^	−1.826^***^
	(0.904)	(0.788)	(0.561)	(0.656)
*Constant*	−12.981^***^	−14.913^***^	−6.311^***^	−6.077^***^
	(3.097)	(2.961)	(1.792)	(2.045)
YEAR	YES	YES	YES	YES
SUBINDUSTRY	YES	YES	YES	YES
N	316	316	316	316

Robust standard deviations are shown in parentheses; ^*^, ^**^, and ^***^ indicate significance at the 10%, 5%, and 1% levels respectively.

#### 4.4.2 Alternative variables test

We conducted a test on the alternative variables of government-enterprise alignment and social contribution. Regarding the calculation of the *GE_Ali* index, the attention level of each enterprise's main disease prevention and control field (*GE_Ali_robust*) is used instead of the original measurement of the comprehensive attention level of each enterprise's disease prevention and control field. The government-enterprise alignment after replacement still has a significant positive correlation with social contribution (α = 0.018, *p* < 0.01). The social dimension score (*WindS*) in WIND's ESG score was selected to replace the dependent variable of social contribution. The regression results still showed a significant positive correlation between government-enterprise alignment on social contribution (α = 0.01, *p* < 0.05). The regression results are shown in Table 9. Hypothesis 1 was once again validated.

**Table 9:** Robustness tests - alternative variables.

**Variables**	**(1)**	**(2)**	**(3)**	**(4)**	**(5)**
	***WindS***	***SC_Total***	***Medical Needs***	***Donation***	***Supply***
*GE_Ali*	0.010^**^				
	(0.005)				
*GE_Ali_robust*		0.018^*^	0.011^**^	0.005	0.003
		(0.010)	(0.005)	(0.005)	(0.003)
*Slack*	1.607^**^	1.066	0.868^***^	0.362	−0.164
	(0.694)	(0.672)	(0.280)	(0.297)	(0.332)
*SOE*	0.003	0.480^*^	−0.154^*^	0.201^*^	0.433^***^
	(0.219)	(0.258)	(0.090)	(0.113)	(0.124)
*Top*1	−0.004	0.003	0.003	0.001	−0.000
	(0.007)	(0.008)	(0.003)	(0.004)	(0.004)
*Size*	0.485^***^	0.506^***^	0.180^***^	0.166^***^	0.159^***^
	(0.102)	(0.100)	(0.039)	(0.042)	(0.047)
*Age*	−0.072^***^	0.016	−0.001	0.011	0.007
	(0.018)	(0.020)	(0.007)	(0.008)	(0.009)
*Lev*	−1.177^**^	−1.354^***^	0.028	−0.657^***^	−0.725^***^
	(0.586)	(0.510)	(0.156)	(0.233)	(0.247)
*Constant*	−4.485^**^	−9.276^***^	−3.918^***^	−2.989^***^	−2.369^**^
	(2.207)	(2.063)	(0.872)	(0.922)	(0.996)
YEAR	YES	YES	YES	YES	YES
SUBINDUSTRY	YES	YES	YES	YES	YES
N	312	316	316	316	316
Adj R^2^	0.154	0.276	0.173	0.170	0.285

Robust standard deviations are shown in parentheses; ^*^, ^**^, and ^***^ indicate significance at the 10%, 5%, and 1% levels respectively.

## 5 Mediating effect

Based on the above analysis, we can expect effective communication and interaction between enterprises and governments to achieve cognitive commonalities and governance structural changes, with more prominent interaction effects, thereby achieving consistency in goals and actions, ultimately producing more behaviors that contribute to society during the public health emergency. Reflexivity requires constant attempts to redesign practices, as well as true openness and reflection for the purpose of mutual learning (29). In order to cope with social crises, enterprises and governments need to maintain transparency and build trust through various channels and forms of communication on a wider range of issues. This study defined the variable of communication between government and enterprises (*Communication*), and determine whether the enterprise has a good communication mechanism with the government by reading texts in corporate CSR reports, ESG reports, and annual reports. A good communication mechanism is not limited to compliant operation and tax payment, but also includes cooperation with the government, participation in policy formulation, providing suggestions and etc. in terms of communication content. It has established stable communication channels or forms, such as regular or daily reports, symposiums between government and enterprises, and frequent communication. If there is a good communication mechanism, score 2; if there is a communication mechanism, score 1; if there is no communication mechanism, score 0.

The following model was constructed to test the mediating effect of the communication mechanism (*Communication*):


(6) $$\begin{array}{r} {SC\text{\_}Total}_{it}=\alpha_{0}+\alpha_{1}{GE\text{\_}Ali}_{it}+\alpha_{i}Control_{it}\\ +SUBINDUSTRY+YEAR+\varepsilon_{it} \end{array}$$

(7) $$\begin{array}{r} Communication_{it}=\alpha_{0}+\alpha_{1}{GE\text{\_}Ali}_{it}+\alpha_{i}Control_{it}\\ +SUBINDUSTRY+YEAR+\varepsilon_{it} \end{array}$$

(8) $$\begin{array}{r} {SC\text{\_}Total}_{it}=\alpha_{0}+\alpha_{1}{GE\text{\_}Ali}_{it}+\alpha_{2}Communication_{it}\\ +\alpha_{i}Control_{it}+SUBINDUSTRY+YEAR+\varepsilon_{it} \end{array}$$

The results of the mediating effect are shown in Table 10. Column (2) shows that a higher level of government-enterprise alignment has a significant positive impact on the establishment of a good communication mechanism between them (α = 0.003, *p* < 0.1). In addition, the coefficients of *GE_Ali* and the communication mechanism in column (3) are both significantly positive, indicating the existence of partial mediating effects. In the context of the public health emergency, government-enterprise alignment can further enhance the social contribution of pharmaceutical enterprises through a good communication mechanism between them.

**Table 10:** The result of mediating effects.

**Variables**	**(1)**	**(2)**	**(3)**
	***SC_Total***	***Communication***	***SC_Total***
*GE_Ali*	0.011^***^	0.003^*^	0.009^**^
	(0.004)	(0.002)	(0.004)
*Communication*			0.488^***^
			(0.160)
*Slack*	0.979	0.648^***^	0.663
	(0.670)	(0.242)	(0.654)
*SOE*	0.483^*^	0.194^**^	0.388
	(0.260)	(0.098)	(0.260)
*Top*1	0.004	−0.004	0.006
	(0.008)	(0.003)	(0.008)
*Size*	0.494^***^	0.209^***^	0.393^***^
	(0.100)	(0.040)	(0.098)
*Age*	0.013	0.017^**^	0.005
	(0.020)	(0.007)	(0.020)
*Lev*	−1.309^**^	−0.448^**^	−1.091^**^
	(0.510)	(0.185)	(0.493)
*Constant*	−8.878^***^	−4.531^***^	−6.667^***^
	(2.083)	(0.863)	(2.052)
YEAR	YES	YES	YES
SUBINDUSTRY	YES	YES	YES
N	316	316	316
Adj R^2^	0.277	0.210	0.301

Robust standard deviations are shown in parentheses; ^*^, ^**^, and ^***^ indicate significance at the 10%, 5%, and 1% levels respectively.

## 6 Discussion

### 6.1 Nature of ownership

The state power, as a source of social recognition, legitimacy, and reputation, influences the social contribution of enterprises. Generally speaking, compared to non-state-owned enterprises, state-owned enterprises are more likely to obtain various resources. Under the impact of major risk events, if the funding chain is relatively stable (30), it is more capable of making contributions to society. Studies have shown that after the COVID-19, the resumption of work and production of state-owned enterprises is significantly better than that of non-state-owned enterprises, and their difficulty in external financing is relatively small. They are willing and able to help private enterprises upstream and downstream of the supply chain (31).

To investigate the impact of state-owned and non-state-owned pharmaceutical enterprises achieving government-enterprise alignment on social contribution during the public health emergency, a heterogeneity test of property rights was conducted. The results are shown in columns (1) and (2) of Table 11. *GE_Ali* has a significant positive impact on social contribution for both state-owned and non-state-owned enterprises in the context of the public health emergency. The significant coefficient of state-owned enterprises (α = 0.020, *p* < 0.1) is greater than that of non-state-owned enterprises (α = 0.006, *p* < 0.1), indicating that state-owned pharmaceutical enterprises achieve higher social contribution through government-enterprise alignment.

**Table 11:** Regression results of heterogeneity test.

**Heterogeneity test**	***SOE***		******Party******		***Capital_city***	
	**Yes**	**No**	**Yes**	**No**	**Yes**	**No**
**Variables**	**(1)**	**(2)**	**(3)**	**(4)**	**(5)**	**(6)**
	***SC_Total***	***SC_Total***	***SC_Total***	***SC_Total***	***SC_Total***	***SC_Total***
*GE_Ali*	0.020^*^	0.006^*^	0.023^**^	0.006	0.011^*^	0.006
	(0.010)	(0.004)	(0.009)	(0.004)	(0.006)	(0.008)
*Slack*	−2.419	0.770	1.202	0.312	1.315	0.021
	(3.157)	(0.722)	(1.905)	(0.767)	(0.949)	(0.986)
*SOE*			−0.002	1.660^**^	0.417	0.190
			(0.323)	(0.837)	(0.331)	(0.475)
*Top*1	0.043^**^	−0.010	0.013	−0.004	0.012	0.002
	(0.021)	(0.008)	(0.018)	(0.009)	(0.010)	(0.014)
*Size*	0.571^*^	0.476^***^	0.652^***^	0.350^**^	0.643^***^	0.296^**^
	(0.325)	(0.116)	(0.195)	(0.139)	(0.136)	(0.144)
*Age*	0.017	0.020	0.027	0.011	−0.012	0.053^*^
	(0.070)	(0.022)	(0.051)	(0.022)	(0.028)	(0.029)
*Lev*	−3.943^**^	−0.449	−3.079^***^	−0.627	−1.366^**^	−0.802
	(1.624)	(0.566)	(1.095)	(0.541)	(0.643)	(0.999)
*Constant*	−10.062	−8.329^***^	−11.832^***^	−5.662^*^	−11.752^***^	−5.281^*^
	(6.213)	(2.419)	(3.868)	(2.974)	(2.782)	(3.067)
YEAR	YES	YES	YES	YES	YES	YES
SUBINDUSTRY	YES	YES	YES	YES	YES	YES
N	75	241	122	194	174	142
Adj R^2^	0.358	0.237	0.324	0.189	0.388	0.110

Robust standard deviations are shown in parentheses; ^*^, ^**^, and ^***^ indicate significance at the 10%, 5%, and 1% levels respectively.

### 6.2 Party organization embedded

Political embeddedness is a typical perspective for explaining the relationship between corporate compliance and government expectations in responding to social issues. Enterprises become important participants and contributors to achieving government missions by following policy guidelines as an effort to shape their political legitimacy (32, 33). With the continuous reform of the relationship between government and enterprises, enterprises are gradually influencing the process of institutional innovation and policy formulation through legal channels, such as relying on grassroots party organizations to embed themselves, the Federation of Industry and Commerce, and other institutionalized methods to participate in institutional design (34). Establishing party organizations in enterprises can create a dual relationship with the government and party organization, further helping enterprises reduce information asymmetry in government-enterprise relationship (35).

The presence or absence of party organizations embedded in a company implies whether the non-economic behavior of party organizations can serve as participants in the allocation of attention to the company's economic behavior (36). In the case of public health emergencies, this kind of corporate behavior favorizing social contribution is particularly important. The cross appointment of members of enterprise's party organizations with members of the board of directors, management, and supervisory board can greatly influence the governance behavior of the enterprise in a “two-way entry” manner (37). Therefore, the number of party committee members among the board of directors, supervisory board, and management executives was selected as the party embedding variable (*Party*) for grouping to explore the differences in the impact of government-enterprise alignment on social contribution in the context of the public health emergency. The results are reported in columns (3) and (4) of Table 11. When the party organization is embedded in corporate governance (Party ≥ 1), the *GE_Ali* significantly affects the social contribution of pharmaceutical enterprises in the context of the public health emergency (α = 0.023, *p* < 0.05). However, when the party organization is not embedded, there is no significant effect. It indicates that when the party organization is embedded in the corporate governance structure, the government-enterprise alignment has a positive promoting effect on the social contribution of pharmaceutical enterprises in the context of the public health emergency.

### 6.3 Geographical location

The theory of geographic distance implies that different organizations can benefit from interaction, which makes their cognition, structure, institutions, and social attributes more common (38, 39). If they have similar features and are closer in spatial attributes, their tendency to interact and achieve a consensus goal is much greater. The geographical advantages of enterprises and governments shorten the time and transportation costs of government investigation, and can promote close communication with enterprises (40). Under the epidemic prevention and control policies, pharmaceutical companies located geographically close to the government can quickly respond to government and social needs, actively engage in social contribution such as material donations, public services, and drug R&D.

Studies have shown that the closer an enterprise is to a big city in terms of geographical location, the more stringent the supervision and review of CSR information disclosure and the stronger the pressure from stakeholders, as well as the imitation pressure from industry competitors, resulting in more outstanding CSR performance (43). The geographical location close to big cities will bring more development resources to enterprises, such as knowledge spillovers, innovation resources, cooperation resources, etc. Correspondingly, external stakeholders such as the government and public media will pay more attention to the enterprise, and its CSR performance becomes particularly important (41). Therefore, we select whether the office location of the enterprise is in the provincial capital city (*Capital_city*) as the heterogeneity test variable of geographical location, and explores the differences in the impact of government-enterprise alignment on social contribution in the context of the public health emergency. The results are reported in columns (5) and (6) of Table 11. When the enterprise is located in the provincial capital city, achieving government-enterprise alignment has a significant positive impact on the social contribution (α = 0.011, *p* < 0.1), while there is no significant impact on enterprises not located in the provincial capital city. This indicates that achieving government-enterprise alignment in pharmaceutical enterprises in the provincial capital city can have a positive promoting effect on social contribution during the public health emergency.

## 7 Conclusion

Under the pandemic situation, pharmaceutical enterprises are placed on the high hope of “good medicine to help the world” and expected to undertake special social responsibility. Meanwhile, governments have also reshaped their responsibilities to address major challenges. In this paper, we demonstrate that good relationship of government and enterprises help them do great contributions to society. Based on the financial and textual data of China's listed pharmaceutical companies and policy data from the official website of the Chinese health-related government departments, we explore the impact of government-enterprise alignment on the social contribution during the public health emergency. Research has found that, firstly, government-enterprise alignment can effectively enhance the social contribution, mainly through increasing the intensity of drug R&D, production and promotion by pharmaceutical companies; secondly, a good communication mechanism can promote government-enterprise alignment, thereby enhancing social contribution; thirdly, state-owned nature, the presence of party organizations embedded, and the location in a provincial capital city can all better achieve a high level of government-enterprise alignment, thereby having a significant positive impact on the social contribution. This study confirms that the Chinese government has made enterprises a part of social governance, which is a global hotspot, through the embedding of party organizations. It also indicates that the government needs to re-recognize its key role in shaping social contribution, especially in distinguishing its responsibilities between normal and emergency situations.

## Data Availability

The original contributions presented in the study are included in the article/supplementary material, further inquiries can be directed to the corresponding author.

## Footnotes

^1^Pfizer/BioNTech, AstraZeneca-Oxford, Janssen, Moderna, Sinopharm and Sinovac.
